# Regulatory Mechanisms of BBX Transcription Factors in Plant Responses to Abiotic Stress and Light Signals

**DOI:** 10.3390/biology15201868

**Published:** 2026-10-16

**Authors:** Pengxiang Chen, Jie Meng, Deyang Kong, Jiahui Cao, Rong Han

**Affiliations:** Higher Education Key Laboratory of Plant Molecular and Environmental Stress Response in Shanxi Province, College of Life Sciences, Shanxi Normal University, Taiyuan 030031, China; cpx2613@126.com (P.C.); 225112044@sxnu.edu.cn (J.M.); kongdeyang@sxnu.edu.cn (D.K.); caojiahui@sxnu.edu.cn (J.C.)

**Keywords:** BBX transcription factors, abiotic stress, drought stress, cold stress, light signaling, regulatory mechanisms

## Abstract

Plants frequently encounter abiotic stresses such as drought, cold, and high light, which severely reduce crop yields. B-box (BBX) transcription factors are emerging as central regulators that link light signaling to stress responses, but their global regulatory networks remain poorly defined. This review synthesizes evidence from *Arabidopsis* and major crops, including tomato, rice, apple, and tobacco, drawing on gene expression analyses, mutant phenotyping, protein interaction assays, and ubiquitination studies. The compiled results show that BBX proteins act as either positive or negative modulators depending on species and stress type. They control stress tolerance by physically interacting with partners like HY5 and PIFs, directly regulating downstream target genes, and undergoing COP1- or MIEL1-mediated proteasomal degradation. Notably, structural differences in the C-terminal region may contribute to their functional divergence, as suggested by domain-swapping experiments. These findings establish BBX proteins as versatile hubs that integrate environmental cues with developmental programs. The conclusions suggest that precise manipulation of specific BBX members or their regulatory domains offers a viable route to enhance broad-spectrum stress resilience in crops, supporting sustainable food production under changing climates.

## Simple Summary

Plants often face harsh conditions, such as drought, cold, and intense light, which substantially reduce crop yields. B-box proteins are special regulatory proteins that act like switches in plants to help them sense and respond to these environmental stresses. This review brings together findings from many studies on these proteins to show how they function in different plant species. Some B-box proteins boost stress tolerance, while others weaken it, and they fine-tune plant responses by interacting with other cellular partners. Importantly, the tail ends of these proteins may influence whether they play a positive or negative regulatory role. These insights provide a solid scientific foundation for breeding new crop varieties that can better withstand harsh weather. In turn, this will help secure food production and reduce farming losses as climate change brings more frequent extreme conditions.

## 1. Introduction

A systematic literature search was conducted in PubMed, Web of Science, and Google Scholar, covering publications from 2000 to 2025, including additional recent studies from 2026. The search terms included “BBX” or “B-box protein” in combination with “abiotic stress”, “drought”, “cold”, “salt”, or “UV-B”. Abiotic stresses, including drought, extreme temperatures, high salinity, and ultraviolet radiation, severely constrain crop yield and geographical distribution, with approximately three-quarters of global harvested areas having experienced yield reduction due to drought from 1983 to 2009 [1]. Extreme temperatures induce membrane lipid peroxidation and protein denaturation, high salinity disrupts ionic homeostasis and osmotic balance, and ultraviolet radiation causes DNA damage and accumulation of reactive oxygen species (ROS)—all of which threaten crop growth and development. Plants employ strategies such as “drought escape” and “drought resistance” to cope with water deficit [2] and respond to other adversities through cold acclimation, salt ion compartmentalization, and UV-B-absorbing compound synthesis. These processes rely on complex signal transduction and transcriptional reprogramming. The B-box (BBX) zinc finger protein family plays critical roles in light signal transduction and abiotic stress responses, participating in the entire plant life cycle from seed germination to flowering and fruiting [3]. However, the roles of BBX proteins in drought, cold, and other stresses; their co-regulatory networks; and their applications in crop stress improvement remain to be elucidated. Unlike recent reviews, this review specifically integrates light signaling with multi-stress responses and highlights the conserved and divergent regulatory mechanisms across diverse crop species.

BBX proteins contain one or two highly conserved B-box domains at the N-terminus, designated B-box1 and B-box2, with some members also possessing a CCT (CONSTANS, CO-like, and TOC1) domain or VP motif. The B-box domain has been shown to mediate specific interaction with the WD40 repeat domain of COP1 [4,5,6]. Based on the number of B-box domains and the presence of the CCT domain, the 32 BBX proteins in *Arabidopsis thaliana* are classified into five groups, with the family being highly conserved across green plants; B-box1 has a single origin, whereas B-box2 independently emerged three times [3,4]. BBX proteins exhibit diverse functions, encompassing photomorphogenesis, flowering regulation, shade avoidance responses, and hormone signal transduction. In tomato, genome-wide expression profiling has identified multiple BBX genes involved in light signaling and fruit ripening [7]. Members within the same family can exert opposing regulatory effects in the same process, and this functional differentiation is closely associated with C-terminal sequence divergence [8]. *BBX8* (*AtBBX8* and AT5G48250), a class II member, serves as a direct target gene of HY5, connecting upstream blue light signals to downstream cold responses. *BBX7* and *BBX8* function as direct HY5 targets that positively regulate freezing tolerance by modulating the expression of a set of cold-responsive genes [9]. For instance, in chrysanthemum, BBX8 accelerates flowering by directly targeting *CmFTL1* [10], and BBX7 interacts with BBX8 to further promote this process [11].

The functional differentiation of BBX proteins into positive and negative regulators has a structural basis. The B-box domain serves as the core module for protein–protein interactions. The CCT domain participates in transcriptional regulation and nuclear localization. The CCT domain of *AtCO* directly binds to the CORE element in the *FT* promoter and indirectly participates in CCAAT-box-mediated transcriptional regulation through interaction with the NF-Y complex [12]. Hd1 (the rice ortholog of *CO*) interacts with OsHAL3 or GHD7 to regulate flowering promotion under short-day conditions and flowering suppression under long-day conditions, respectively [13,14]. The VP motif is a characteristic feature of class III BBX proteins (*BBX14–17*), which lack the CCT domain. Deletion of the VP motif leads to loss of interaction with COP1, altering protein stability and consequently affecting regulatory functions [5].

## 2. Expression Regulation Patterns of BBX Genes Under Abiotic Stress

In this section, the regulatory mechanisms of BBX proteins under three main abiotic stresses are elaborated sequentially from three perspectives: drought stress, cold stress, and light signaling and light-associated stress responses.

### 2.1. Drought Stress

Drought constitutes the primary abiotic stress factor limiting crop productivity. BBX proteins participate in drought stress responses through abscisic acid (ABA)-dependent or -independent pathways, exhibiting bidirectional functions as either positive or negative regulators across different plant species.

In *Arabidopsis thaliana*, *AtBBX1* (CONSTANS) mediates the GI-CO-FT module that enables the ABA-dependent “drought escape” response, whereby flowering is promoted under drought conditions to complete the life cycle [15]. This finding reveals the cross-talk between flowering regulation pathways and drought response pathways. *AtBBX5* (also known as *AtCOL4*) positively regulates abiotic stress tolerance, particularly salt stress responses, through the ABA-dependent signaling pathway. *Atbbx5* mutants exhibit heightened sensitivity to ABA and salt stress, whereas *AtBBX5*-overexpressing plants display enhanced tolerance to these stresses [16]. Further investigation revealed that AtBBX5 regulates the expression of ABA biosynthesis-related genes (*ABA1* and *NCED3*) and stress-responsive genes (*RD29A* and *RD29B*). Conversely, *AtBBX21* negatively regulates ABI5 expression by interfering with HY5 binding to the *ABI5* promoter, thereby promoting seed germination [17].

In crop species, the drought regulatory functions of BBX genes exhibit species-specific characteristics. In tomato (*Solanum lycopersicum*), a study identified natural variation in *SlBBX18* as a key determinant of drought sensitivity in cultivated tomato. Wild tomato (*Solanum pennellii*) encodes the full-length SpBBX18 protein (containing the C-terminus), whereas cultivated tomato (*Solanum lycopersicum*) contains a SNP-265 (C→T) that introduces a premature stop codon, producing the C-terminally truncated SlBBX18 protein [18]. *SlBBX18* functions as a negative regulator of drought tolerance. The full-length *SpBBX18^CC^* protein maintains ROS homeostasis and drought tolerance, whereas the truncated *SlBBX18^TT^* protein in cultivated tomato leads to reduced antioxidant defense, ROS accumulation, and increased drought sensitivity, revealing the critical role of BBX natural variation in crop domestication [18]. *SlBBX19* and *SlBBX24* are inducible under drought, PEG, and heat stress, and family members including *SlBBX5*, *7*, *15*, *17*, *20*, *22*, and *24* localize to the nucleus; however, the specific functional mechanisms of *SlBBX19* and *SlBBX24* in drought response require further investigation [19]. Additionally, *SlBBX20* may contribute to saline–alkali stress responses by regulating the expression of stress-related genes (*SlP5CR*, *SlHsp21.6A*, and *SlHsp26.2*) [20].

In sweet potato *(Ipomoea batatas*), the *IbBBX28* gene was cloned and functionally characterized. IbBBX28 contains a conserved B-box domain, and its expression is induced by drought stress [21]. Heterologous overexpression of *IbBBX28* in *Arabidopsis* resulted in significantly reduced activities of protective enzymes (SOD, POD, and CAT) and elevated malondialdehyde (MDA) content in transgenic plants under drought stress, whereas the *Arabidopsis* mutant *bbx28* exhibited the opposite trend, indicating that *IbBBX28* negatively regulates drought tolerance. Furthermore, yeast two-hybrid and BiFC assays verified the interactions of IbBBX28 with IbHOX11 and IbZMAT2, suggesting that IbBBX28 may regulate stress responses through interaction with these proteins.

In wheat (*Triticum aestivum*), 96 *TaBBX* genes were identified through genome-wide analysis [22]. Promoter analysis revealed that *TaBBX* genes contain abundant stress- and hormone-responsive cis-elements, including ABA-responsive elements (ABREs) and drought-responsive elements (MBS), predicting their potential functions in abiotic stress responses [22,23].

In pepper (*Capsicum annuum*), 24 *CaBBX* genes were identified. Expression analysis revealed that *CaBBX4*, *CaBBX5*, and *CaBBX6* are upregulated under PEG-simulated drought stress, with *CaBBX4* being strongly induced, suggesting that these *CaBBX* members may be involved in drought stress responses in pepper [24].

In tobacco (*Nicotiana tabacum*), 43 *NtBBX* genes were identified from the genome and classified into five subfamilies [25]. Promoter analysis suggested that the promoter regions of *NtBBX* genes contain abundant light-responsive elements, hormone-responsive elements (such as MeJA, ABA, GA, and SA response elements), and stress-responsive elements (such as low temperature, drought, and defense response elements). Transcriptome analysis revealed that under dehydration stress, the expression of 28 *NtBBX* genes changed significantly, with all members of subfamilies I and III being significantly downregulated [25]. Exogenous melatonin treatment partially reversed the dehydration-induced expression changes in multiple *NtBBX* genes, suggesting that these genes may be associated with dehydration stress responses through the melatonin pathway. Together, these results suggest that tobacco BBX genes may be broadly responsive to multiple abiotic stresses at the transcriptional level, but their functional roles require further validation [25].

In apple (*Malus domestica*), *MdBBX1* is significantly upregulated under multiple abiotic stresses and ABA treatment, and its overexpression in *Arabidopsis* enhances tolerance to salt and drought stress [26]. *MdBBX7* positively regulates drought tolerance. *MdBBX7* directly activates the expression of drought-responsive genes including *MdERF1*, *MdERD15*, and *MdGLK1*; however, under normal water conditions, MdBBX7 protein is degraded through the 26S proteasome pathway mediated by the E3 ubiquitin ligase MdMIEL1 [27]. Apple plants overexpressing *MdBBX7* exhibited significantly enhanced drought stress tolerance. Similarly, *MdBBX22* negatively regulates ABA-induced leaf senescence through a dual mechanism: it interacts with MdABI5 protein to inhibit its transcriptional activity on the chlorophyll degradation genes *MdNYE1* and *MdNYC1* and also interacts with *MdHY5* to interfere with its transcriptional activation of *MdABI5* [28].

In rice (*Oryza sativa*), *OsBBX6*, a member of the rice BBX family, contains a drought-responsive element (MBS) in its promoter region. Expression analysis revealed that it is upregulated by low temperature but suppressed by drought and salt stress, suggesting its potential involvement in cold, drought, and salt stress responses at the transcriptional level [29]. *OsBBX7* is expressed at both the seedling and heading stages in rice; its expression is upregulated by drought, cold, salt, and hormone treatments (GA, SA, and MeJA), and heavy metals (Fe, Ni, Cr, and Cd) also significantly affect its transcription level [30]. *OsBBX9* expression is downregulated by ABA treatment but upregulated by Ni treatment, and some BBX genes show significant expression changes under multiple abiotic stresses; however, the specific function of *OsBBX9* under drought stress requires further validation [30]. *Ghd2,* a rice CONSTANS-like gene, negatively regulates drought tolerance. OsGHD2-overexpressing plants accelerate drought-induced leaf senescence through upregulation of senescence-associated genes (*OsSGR1* and *OsPAO*) [31]. OsBBX11 positively regulates ion homeostasis; its loss leads to increased Na^+^ accumulation and decreased K^+^/Na^+^ ratio under salt stress [32]. In chrysanthemum (*Chrysanthemum morifolium*), *CmBBX19* interacts with *CmABF3* to inhibit ABA responses, negatively regulating drought tolerance [33]. Collectively, these studies demonstrate that BBX proteins exert bidirectional regulatory functions in drought stress responses, involving ABA signaling, leaf senescence, ion homeostasis, and multiple other pathways.

**Figure 1:**
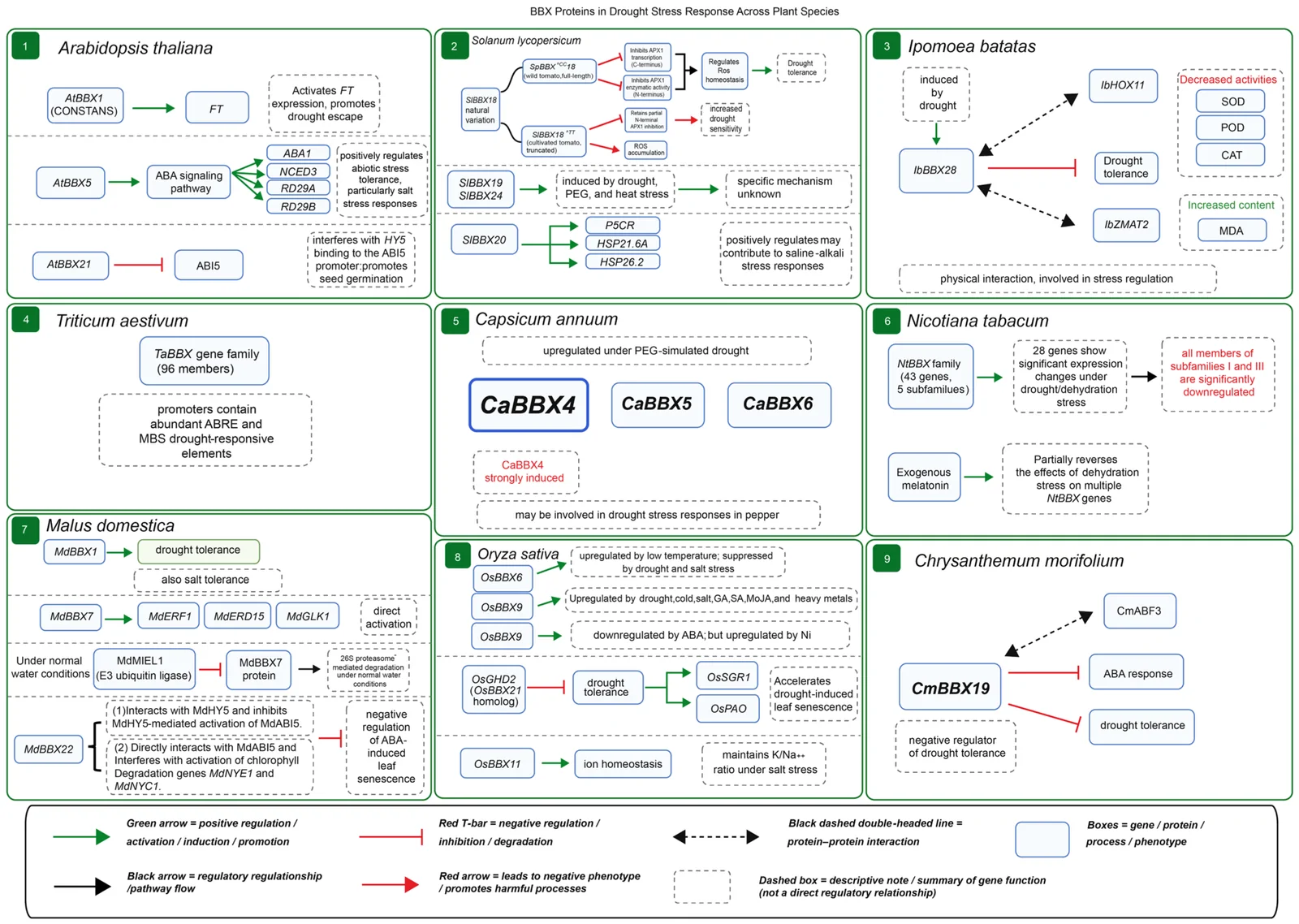
Regulatory networks of BBX proteins in drought stress responses across different plant species. 1. *Arabidopsis thaliana*; 2. *Solanum lycopersicum*; 3. *Ipomoea batatas*; 4. *Triticum aestivum*; 5. *Capsicum annuum*; 6. *Nicotiana tabacum*; 7. *Malus domestica*; 8. *Oryza sativa*; 9. *Chrysanthemum morifolium*. Green arrows indicate positive regulation/activation; red T-bars indicate negative regulation/inhibition; red solid arrows indicate leads to negative phenotype; black solid arrows indicate regulatory relationships; black dashed double-headed lines indicate protein-protein interactions; dashed boxes indicate descriptive notes/summary of gene function. In the boxes, red text denotes decreased activity or content, and green text denotes increased activity or content. Boxes represent genes, proteins, processes, or phenotypes. Abbreviations: ABA, abscisic acid; ROS, reactive oxygen species; SOD, superoxide dismutase; POD, peroxidase; CAT, catalase; MDA, malondialdehyde.

The regulatory patterns of BBX proteins in drought stress response across different plant species are summarized in Figure 1.

### 2.2. Cold Stress

Low-temperature stress severely constrains plant geographical distribution and crop yield. The mechanisms by which BBX proteins function in cold stress responses have been systematically elucidated in *Arabidopsis* and progressively validated in crop species.

In *Arabidopsis*, the complete pathway of the *CRY2-COP1-HY5-BBX7/8* module regulating blue light-dependent cold acclimation has been systematically elucidated [9]. The core findings are as follows: blue light induces CRY2 phosphorylation, and cold stress promotes the stability of phosphorylated CRY2; phosphorylated CRY2 interacts with COP1, competitively inhibiting COP1 binding to HY5, thereby stabilizing HY5 protein; stabilized HY5 directly targets the *BBX7* and *BBX8* genes, activating their transcription; and BBX7 and BBX8 proteins positively regulate *COR* gene expression and enhance freezing tolerance mainly independently of the CBF pathway [9]. This study established a complete signaling cascade linking the blue light receptor CRY2, E3 ubiquitin ligase COP1, transcription factor HY5, and BBX proteins, revealing the molecular mechanism integrating light and cold signals.

In tomato, the cold stress regulatory network exhibits multilayered features. *SlBBX17* positively regulates CBF-dependent cold tolerance through phosphorylation mediated by mitogen-activated protein kinases SlMPK1/2 [34]. *SlBBX31* is transcriptionally activated by SlHY5 [35]. A 27 bp InDel in its promoter, present in wild tomato accessions, impairs SlHY5-mediated transactivation of *SlBBX31*, resulting in reduced *SlBBX31* expression and impaired cold tolerance. The absence of this InDel in cultivated tomato is associated with enhanced cold tolerance, and *SlBBX31* was possibly selected during tomato domestication for cold tolerance regulation [35]. *SlBBX7*, *SlBBX9*, and *SlBBX20* positively regulate cold tolerance; their silenced plants exhibit decreased non-photochemical quenching (NPQ) under cold stress, leading to excess photon energy and electron flow, over-reduction in electron carriers, and damage to the photosystem [36]. This study demonstrates that BBX proteins enhance cold tolerance by protecting photosynthetic apparatus integrity.

In potato (*Solanum tuberosum*), *StBBX14* expression was found to be significantly induced by cold stress, peaking at 1 h and then gradually declining. The StBBX14 protein is predominantly localized to the nucleus. Potato lines overexpressing *StBBX14* exhibited less leaf damage and significantly reduced electrolyte leakage under freezing stress (−2 to −6 °C), indicating that *StBBX14* positively regulates cold tolerance in potato. Transcriptomic analysis identified 2449 and 6274 differentially expressed genes; WGCNA generated 14 co-expression modules, of which 4 were associated with cold-related genes. GO enrichment analysis revealed that pathways including photosynthesis, cell wall, and transcriptional regulatory activity were significantly enriched in *StBBX14*-overexpressing lines, providing insights into the potential involvement of *StBBX14* in cold acclimation pathways. However, the direct downstream targets of *StBBX14* remain to be identified [37].

In pepper (*Capsicum annuum*), the expression patterns of BBX family members under multiple abiotic stresses have been systematically analyzed [24]. Using qRT-PCR, six *CaBBX* genes (*CaBBX3*, *CaBBX4*, *CaBBX5*, *CaBBX6*, *CaBBX13*, and *CaBBX20*) were examined under cold, heat, salt, and drought treatments at 3, 6, and 12 h [24]. Under cold stress, four of the six genes were upregulated, whereas *CaBBX13* and *CaBBX20* were strongly induced at 3 h but rapidly downregulated thereafter.

In melon (*Cucumis melo*), 19 *BBX* gene family members were identified through genome-wide analysis [38]. Under various abiotic and biotic stress conditions, certain *CmBBX* genes (including *CmBBX3*, *CmBBX5*, *CmBBX2*, *CmBBX18*, *CmBBX15*, and *CmBBX11*) exhibited significant differential expression, suggesting their potential involvement in melon stress responses at the transcriptional level.

In tobacco (*Nicotiana tabacum*), transcriptomic analysis revealed that several *NtBBX* genes exhibited differential expression under cold stress, with subfamily IV members being the most prominent [25]. However, a separate functional study of the 49-member tobacco BBX family identified two closely related group II members with contrasting roles. *NtBBX11* positively regulates cold tolerance; it is upregulated by cold induction, and its overexpression enhances cold tolerance in transgenic tobacco through regulation of *CBFs*, *LEA14*, and *LTI65* genes, as well as ROS levels [39]. Conversely, *NtBBX9* negatively regulates cold tolerance; overexpressing plants are cold-sensitive, while RNAi-silenced lines exhibit enhanced cold tolerance through de-repression of CBF expression [39]. Similarly, *NtBBX46* negatively regulates cold tolerance; it localizes to the nucleus, and silenced lines show reduced MDA and ROS levels and enhanced cold tolerance through transcriptional repression of *NtCBF5* [40].

In apple, *MdBBX20* expression responds to low-temperature stress (14 °C), a process requiring MdbHLH3, which directly binds to the *MdBBX20* promoter [41]. *MdBBX37* functions as a positive regulator of JA-mediated cold tolerance by binding to and activating the *MdCBF1/4* promoters, while also interacting with the MdICE1 protein to enhance its transcriptional activity on *MdCBF1*. MdJAZ1/2 proteins interact with MdBBX37, inhibiting its transcriptional activity on CBF promoters and disrupting the MdBBX37-MdICE1 interaction; the E3 ubiquitin ligase MdMIEL1 attenuates cold resistance by mediating ubiquitination and degradation of MdBBX37. Together, these components constitute the JAZ-BBX37-ICE1-CBF regulatory cascade [42].

Collectively, the mechanisms by which BBX proteins function in cold stress responses can be summarized as follows: (i) in some cases, *BBX* genes act downstream of light-signaling components such as HY5, thereby linking light signals to cold responses; (ii) BBX proteins regulate CBF-dependent and/or CBF-independent *COR* gene expression; and (iii) certain BBX members alleviate cold-induced photoinhibition by protecting photosynthetic apparatus integrity.

**Figure 2:**
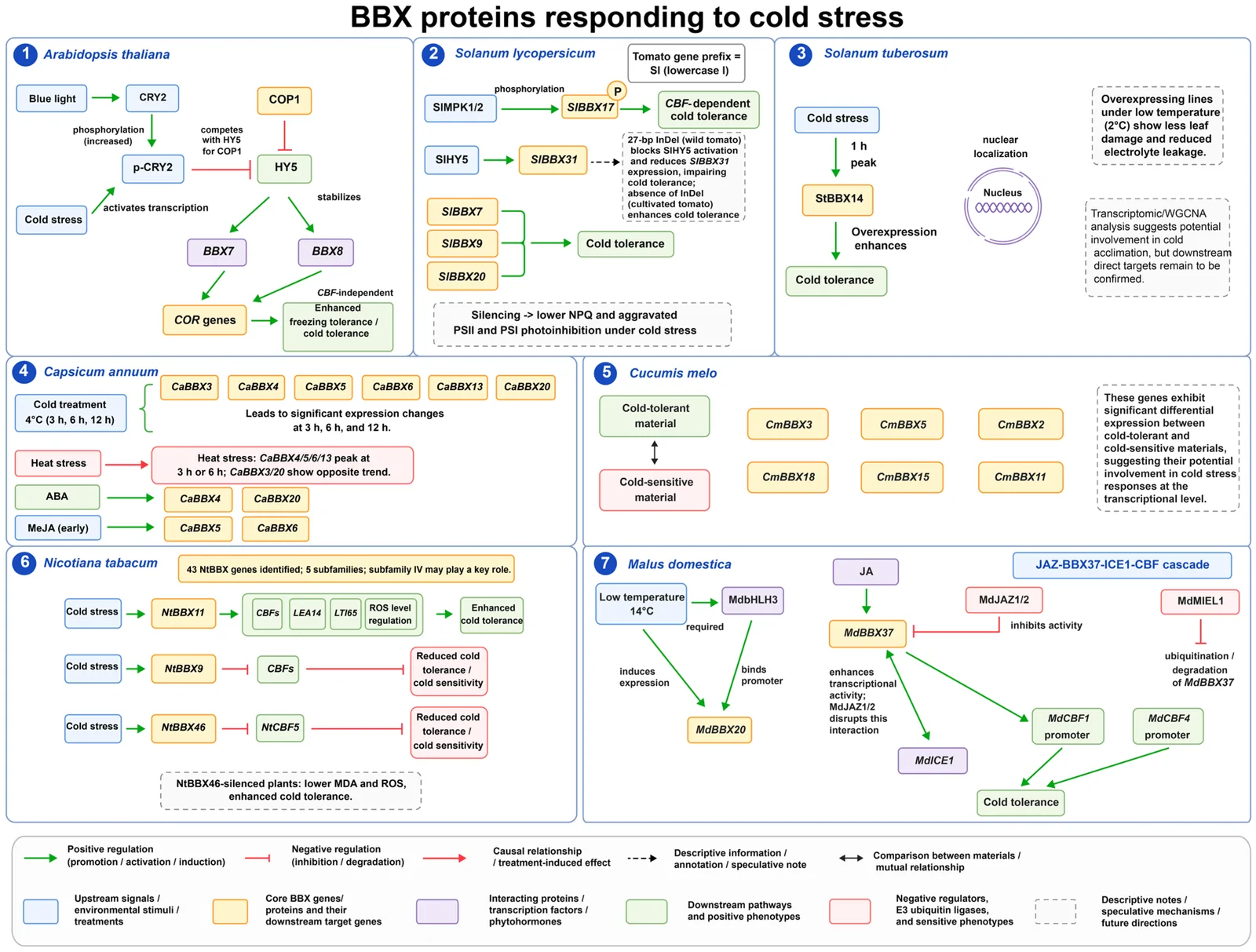
Regulatory patterns of BBX proteins responding to cold stress across different plant species. 1. *Arabidopsis thaliana*; 2. *Solanum lycopersicum*; 3. *Solanum tuberosum*; 4. *Capsicum annuum*; 5. *Cucumis melo*; 6. *Nicotiana tabacum*; 7. *Malus domestica*. Green arrows indicate positive regulation; red T-bars indicate negative regulation; red solid arrows indicate causal relationship; black dashed arrows indicate descriptive notes; black double-headed arrows indicate comparison between materials. Boxes represent genes, proteins, processes, or phenotypes. Abbreviations: CBF, C-repeat binding factor; COR, cold-responsive; ROS, reactive oxygen species; NPQ, non-photochemical quenching; PSII/PSI, photosystem II/I. Conclusion: BBX proteins positively or negatively regulate cold tolerance through CBF-dependent and CBF-independent pathways, as well as via protein-protein interactions and ubiquitination.

The regulatory patterns of BBX proteins in cold stress response across different plant species are summarized in Figure 2.

### 2.3. Light Signaling and Light-Associated Stress Responses

Light serves as both the energy source for photosynthesis and a critical environmental signal regulating plant development. However, insufficient light (shading), altered light quality (low R/FR ratio), or high-energy ultraviolet radiation (UV-B) can all impose stress on plants. BBX proteins perform central regulatory functions in light signaling and associated stress responses, characteristically exhibiting “bidirectional positive–negative” regulation.

#### 2.3.1. Photomorphogenesis and Shade Avoidance

##### Photomorphogenesis

In *Arabidopsis* photomorphogenesis, *BBX9* functions as a positive regulator of light signaling. Mechanistically, phyB promotes BBX9 protein stability under light; *BBX9* suppresses the transcriptional activation activity of PIFs; PIFs bind to the *BBX9* promoter to inhibit its transcription, forming a negative feedback loop; and *BBX21* activates *BBX9* transcription, forming a positive feedback loop. *BBX9* promotes photomorphogenesis through dual feedback loops with PIFs and BBX21 [43]. *AtBBX21* and *AtBBX22* are positive regulators. AtBBX21 directly binds to the T/G-box in the HY5 promoter to activate its transcription, and its protein is targeted for 26S proteasome degradation by COP1 [44]. AtBBX22 interacts with HY5 protein to enhance its transcriptional activity, and it is also regulated by COP1-mediated ubiquitination degradation [45,46]. AtBBX23 similarly interacts with HY5, and both are directly transcriptionally activated by PIF1 and PIF3, forming a PIF1/PIF3-HY5-BBX23 transcriptional cascade that promotes photomorphogenesis [47]. Conversely, *AtBBX24* and *AtBBX25* are negative regulators; they interact with HYH (HY5 homolog) and antagonize HY5 function indirectly through HYH depletion. BBX25 has also been shown to directly interact with HY5 [46], while BBX24/25 together may form inactive complexes with HYH to inhibit photomorphogenesis [48].

In tomato, genome-wide identification followed by expression profiling identified several BBX genes as light- and ripening-responsive [7]. Among them, *SlBBX4* positively regulates photomorphogenesis under red light; its interaction with COP1 suggests that its protein stability may be regulated by ubiquitination degradation [49]. *SlBBX20* is a positive regulator of light signaling, regulating photomorphogenesis through suppression of the SlPIF4–auxin interaction; additionally, *SlBBX20* cooperates with SlHY5 to upregulate the expression of flavonoid biosynthesis-related genes, participating in anthocyanin accumulation [50]. It has been reported that *SlBBX26* influences gibberellin synthesis and signaling through regulation of SlPIF4, thereby modulating vegetative growth and flowering; simultaneously, *SlBBX26* regulates fruit growth-related genes, chloroplast ultrastructure, and metabolism. Furthermore, the SlBBX26-SlPIF4 heterodimer coordinately regulates gibberellin and auxin signaling cascades, triggering fruit ripening initiation [51].

In rice, it has been reported that *OsBBX14* positively regulates photomorphogenesis under blue light: overexpression results in seedling dwarfing (reduced cell length), while knockout increases plant height. Mechanistically, OsBBX14 interacts with OsCRY2, binds the T/G-box in the *OsHY5L1* promoter, and is associated with upregulation of *OsHY5L1*, cell wall organization, and dwarfing-related genes [52]. It has also been reported that *OsBBX2* is highly expressed in roots, exhibits circadian transcriptional rhythms, and possesses transcriptional repression activity. In the context of non-functional or weakly functional *Ghd7* and *PRR37*, *OsBBX2* overexpression delays heading date under long-day conditions. *OsBBX2* interacts with Hd1 to co-repress Hd3a transcription [53].

In apple, *MdBBX21* is expressed under light treatment and localizes to the nucleus, and overexpression increases anthocyanin accumulation in apple callus and *Arabidopsis*. It can bind to and activate the promoters of *MdHY5*, *MdBBX20*, and *MdBBX22-1/2*, while also interacting with MdHY5 protein to synergistically enhance *MdMYB1* promoter activity [54].

In the C4 model plants foxtail millet (*Setaria italica*) and green foxtail (*Setaria viridis*), 33 BBX family members were systematically identified (16 *SiBBX*; 17 *SvBBX*) and classified into subfamilies I, III, and IV. Multiple *SiBBX* genes exhibited pronounced circadian rhythmic expression under long-day conditions, such as *SiBBX1* and *SiBBX3* (homologs of *AtCO*) peaking at midday (13:00), and four tested SiBBX proteins (SiBBX3, SiBBX5, SiBBX8, and SiBBX13) localized to the nucleus, suggesting their potential involvement in light-responsive transcriptional regulation. *SiBBX5* and *SiBBX16* displayed opposite circadian expression patterns, implying functional differentiation within the BBX family [55]. Furthermore, different light qualities (red and blue light) significantly affected the expression levels of multiple *SiBBX* and *SvBBX* genes; for example, *SiBBX2* and *SiBBX13* were strongly induced under red/blue light, indicating that they may be important components of light signaling pathways [55].

In yam (*Dioscorea opposita*), *DoBBX2/DoCOL5* and *DoBBX8/DoCOL8* exhibited opposite expression patterns during microtuber formation; both showed the highest expression in leaves and displayed photoperiod-responsive expression patterns. Heterologous overexpression of both *DoBBX2* and *DoBBX8* accelerated tuber formation in potato under short-day conditions, but only *DoBBX8* overexpression enhanced tuber induction under dark conditions [56]. These results suggest that BBX genes may serve as regulatory nodes connecting photoperiod signals to underground storage organ development.

In tobacco (*Nicotiana tabacum*), light-responsive cis-acting elements (such as G-box, GT1-motif, and TCT-motif) were widely distributed in the promoter regions of the 43 *NtBBX* genes, suggesting that these *NtBBX* genes may be involved in light signal transduction pathways at the transcriptional level [25]. Evolutionary analysis further revealed that *NtBBX18*–*NtBBX35* cluster with known photomorphogenesis regulators in *Arabidopsis*—the positive regulators *AtBBX21/AtBBX22* and the negative regulators *AtBBX24/AtBBX25*—in the same group, suggesting their potential involvement in tobacco photomorphogenesis [25].

##### Shade Avoidance

In shade avoidance responses (low R/FR ratio), BBX proteins similarly exhibit functional differentiation. *AtBBX21* positively regulates shade avoidance-related gene expression under short-term shading but negatively regulates shade avoidance responses under long-term shading through downregulation of auxin, ethylene, and other hormone signaling pathways. Plants overexpressing *AtBBX21* show inhibited hypocotyl elongation under shade conditions; thus, this protein is generally considered a negative regulator of shade avoidance [57]. *AtBBX24* positively regulates shade avoidance responses by interacting with DELLA proteins to relieve their inhibition of PIF4, thereby promoting cell elongation [58]. Notably, *AtBBX29* functions as a negative regulator of photomorphogenesis [59], while *AtBBX28* positively regulates shade avoidance responses by promoting hypocotyl elongation under shade [60]. *AtBBX28* perceives reduced red light through the phytochrome system and accumulates in the nucleus in a COP1-dependent manner under shade conditions, promoting hypocotyl elongation. *AtBBX28* upregulates the expression of transcription factors and auxin-related genes, sustaining shade-avoidance responses under prolonged shade [60].

In maize, *ZmDBB2* is a core regulator of shade avoidance responses: under low R/FR conditions, *ZmPIF4* activates *ZmDBB2* expression, and *ZmDBB2* interacts with ZmPIF4 to enhance the transcriptional activation of cell elongation-related genes (such as *ZmEXPA1*); under high R/FR conditions, ZmDBB2 interacts with ZmHY5 to inhibit expression of the GA degradation gene *ZmGA2ox4*, thereby positively regulating maize plant height through the gibberellin pathway [61].

The positive and negative regulatory mechanisms of BBX proteins in photomorphogenesis and shade avoidance reveal the general principle that members of the same family achieve functional differentiation through different interacting partners depending on temporal context.

#### 2.3.2. UV-B Stress

UV-B radiation induces photomorphogenic responses and activates stress defense pathways. BBX proteins play important regulatory roles in UV-B signal transduction.

In *Arabidopsis*, *AtBBX24* functions as a negative regulator of the UV-B signaling pathway. Under UV-B irradiation, *AtBBX24* expression is upregulated, and its protein accumulation depends on COP1; the AtBBX24 protein interacts with COP1 and directly interacts with HY5 to inhibit its transcriptional activity, thereby negatively regulating UV-B-induced photomorphogenesis, although *BBX24* also has HY5-independent effects. *AtBBX24* has been proposed to act in a UV-B feedback module that may also include RCD1 and RUP1/RUP2 [62]. RCD1 interacts with the BBX24 protein, and RCD1 has been reported to influence STO/BBX24 transcript levels under UV-B, although a direct transcriptional mechanism remains unproven. *AtBBX31* plays a positive regulatory role in UV-B signaling: under white light, BBX31 negatively regulates photomorphogenesis in an HY5-independent manner; however, under high-dose UV-B irradiation, BBX31 switches to a positive regulator, promoting the accumulation of UV-protective flavonoids and phenolic compounds. The specific role of BBX31 under low-dose UV-B requires further experimental validation. Bioinformatics analysis and qRT-PCR validation showed that *AtBBX1*, *AtBBX7*, *AtBBX20*, *AtBBX25*, and *AtBBX32* are significantly upregulated under UV-B radiation, suggesting that these genes may be involved in UV-B responses at the transcriptional level [63]. In *Arabidopsis*, in addition to the BBX members mentioned above, *BBX5* was also identified as a novel regulator of UV-B tolerance; the HY5-BBX5-ANS transcriptional regulatory axis mediates UV-B-induced anthocyanin accumulation [64]. *BBX22* is rapidly induced under high-intensity UV-B, exerting multiple protective effects through promoting antioxidant accumulation and DNA repair gene expression [65]. *BBX20-22* function as co-activators of HY5 in UVR8-mediated UV-B signaling, though other unknown HY5 co-activators exist during the early response phase [66].

In crop species, this regulatory module exhibits evolutionary conservation. In tomato, *SlBBX20* and *SlBBX21* interact with SlHY5 and bind to the *SlHY5* promoter to activate its transcription, forming the SlBBX20/21-SlHY5 module that activates SlHY5 transcription under UV-B, with SlHY5 in turn outcompeting SlBBX20/21 for promoter binding and attenuating its own transcription through an autoregulatory negative feedback loop [67]. *MdBBX20* expression in apple is significantly induced by UV-B, promoting anthocyanin biosynthesis under UV-B conditions; it specifically binds to G-box elements in target gene promoters and interacts with MdHY5 to significantly enhance *MdMYB1* promoter activity, thereby integrating low temperature- and UV-B-induced anthocyanin accumulation [41]. *MdBBX22* positively regulates UV-B-induced anthocyanin biosynthesis by enhancing the binding of MdHY5 to its target gene promoters [68]. Collectively, these studies demonstrate that the BBX-HY5 transcriptional regulatory module is conserved in UV-B responses [66,67].

**Figure 3:**
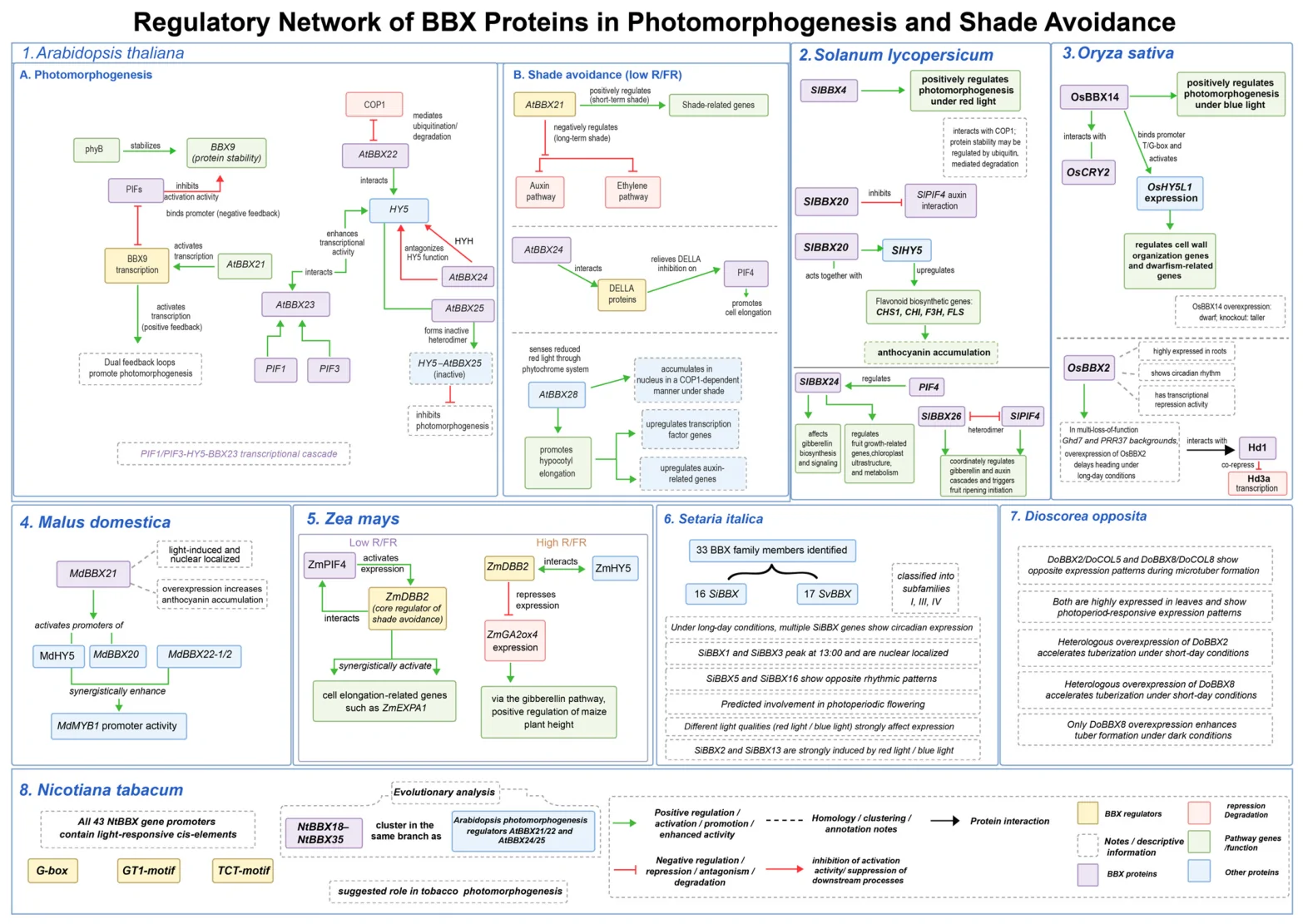
Regulatory network of BBX proteins in photomorphogenesis and shade avoidance across different plant species. 1. *Arabidopsis thaliana* (photomorphogenesis and shade avoidance); 2. *Solanum lycopersicum*; 3. *Oryza sativa*; 4. *Malus domestica*; 5. *Zea mays*; 6. *Setaria italica*; 7. *Dioscorea opposita*; 8. *Nicotiana tabacum*. Green arrows indicate positive regulation/activation; red T-bars indicate negative regulation/repression/degradation; red solid arrows indicate inhibition of activation activity; black dashed arrows indicate homology/clustering/annotation notes; black double-headed arrows indicate protein-protein interaction/comparison between materials. Boxes represent genes, proteins, pathways, or phenotypes. Abbreviations: phyB, phytochrome B; PIFs, phytochrome-interacting factors; COP1, constitutive photomorphogenic 1; HY5, elongated hypocotyl 5; R/FR, red to far-red light ratio; DELLA, DELLA proteins; NPQ, non-photochemical quenching; PSII/PSI, photosystem II/I; JA, jasmonic acid; ABA, abscisic acid. Conclusion: BBX proteins exhibit functional differentiation in photomorphogenesis and shade avoidance through dual feedback loops, protein-protein interactions, phytohormone signaling, and ubiquitin-mediated degradation. Notably, *AtBBX28* acts as a negative regulator of photomorphogenesis but promotes hypocotyl elongation in shade avoidance.

**Figure 4:**
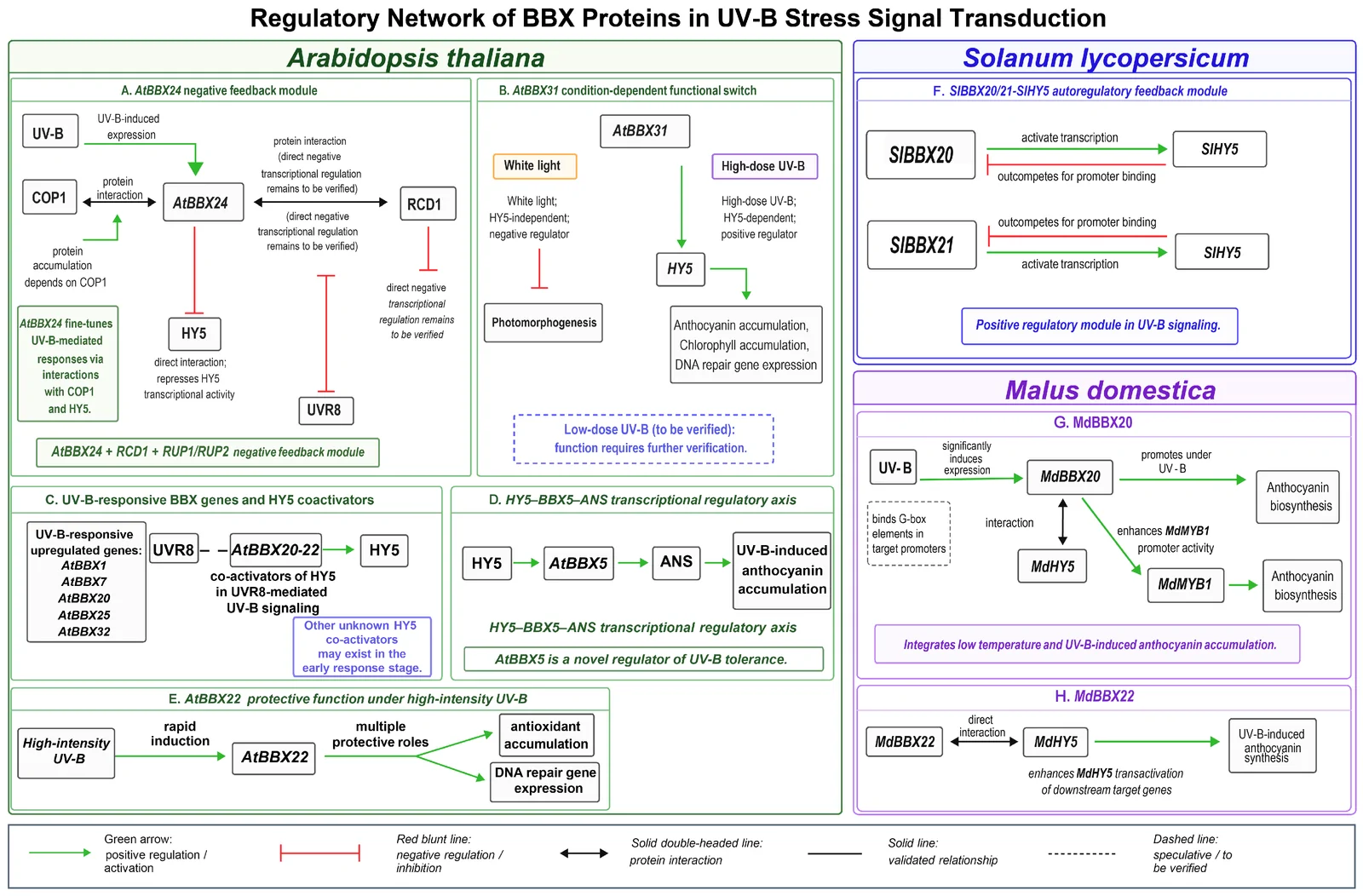
Regulatory network of BBX proteins in UV-B stress signal transduction across different plant species. (**A**) *AtBBX24* negative feedback module in *Arabidopsis thaliana*; (**B**) *AtBBX31* condition-dependent functional switch; (**C**) UV-B-responsive *BBX* genes and HY5 coactivators; (**D**) HY5-BBX5-ANS transcriptional regulatory axis; (**E**) *AtBBX22* protective function under high-intensity UV-B; (**F**) *SlBBX20/21*-SlHY5 autoregulatory feedback module in *Solanum lycopersicum*; (**G**) *MdBBX20* module and (**H**) *MdBBX22* module in *Malus domestica*. Green arrows indicate positive regulation/activation; red blunt lines indicate negative regulation/inhibition; solid double-headed lines indicate protein-protein interactions; solid lines indicate validated regulatory relationships; dashed lines indicate speculative or yet-to-be-validated relationships. Boxes represent genes, proteins, processes, or phenotypes. Abbreviations: UV-B, ultraviolet-B; COP1, constitutive photomorphogenic 1; HY5, elongated hypocotyl 5; UVR8, UV resistance locus 8; RUP1/2, repressor of UV-B photomorphogenesis 1/2; RCD1, radical-induced cell death 1. Conclusion: The BBX-HY5 transcriptional regulatory module is conserved in UV-B responses, with BBX proteins functioning as either positive or negative regulators depending on the light conditions and interacting partners.

The regulatory network of BBX proteins in photomorphogenesis and shade avoidance is shown in Figure 3. The regulatory network of BBX proteins in UV-B stress signal transduction is presented in Figure 4.

## 3. Mechanistic Analysis of Positive and Negative Regulation by BBX Proteins

The previous sections described the functions of BBX family members under various stress conditions, showing a clear pattern: members within the same family exhibit bidirectional positive and negative regulatory characteristics when controlling the same trait. This section provides a systematic analysis from three perspectives: phylogenetic classification, conserved sequence features, and mechanisms of functional differentiation.

### Classification and Functions of Positive and Negative Regulators

As summarized from the literature mentioned above, BBX proteins exhibit pronounced functional differentiation in stress responses and photomorphogenesis.

Positive regulators identified in *Arabidopsis* include *AtBBX1* (CO), which positively regulates flowering [15]; *AtBBX5*, which enhances salt and osmotic stress tolerance [16] and promotes UV-B resistance through anthocyanin biosynthesis [64]; *AtBBX9* and *AtBBX23*, both of which promote photomorphogenesis [43,47]; and *AtBBX7* and *AtBBX8*, which function as direct targets of HY5 and positively regulate cold tolerance through a mainly CBF-independent pathway [9]. Additionally, *AtCOL3* (later designated *BBX4*) acts as a positive regulator of red light signaling and root growth [69]. *AtBBX20*, *AtBBX21*, and *AtBBX22* form a transcriptional activation module with HY5 to positively regulate photomorphogenesis and anthocyanin synthesis [70], with *AtBBX22* also promoting photoprotection and DNA repair under UV-B [65], and *AtBBX31* plays a positive regulatory role in UV-B signaling [71]. In crop species, tomato *SlBBX17* positively regulates cold/heat stress [34,72]; *SlBBX31* positively regulates cold tolerance [35]; *SlBBX7* and *SlBBX9* positively regulate cold tolerance [36]; *SlBBX4* positively regulates photomorphogenesis [49]; and *SlBBX21* cooperates with *SlBBX20* to exert positive regulatory functions in UV-B signaling [67]. In apple, *MdBBX1* positively regulates drought tolerance [26], *MdBBX37* positively regulates JA-mediated cold tolerance [42], and *MdBBX7* positively regulates drought tolerance [27]. In rice, *OsBBX11* affects Na^+^/K^+^ transport and positively regulates salt tolerance [32]; *OsBBX14* positively regulates photomorphogenesis under blue light [52]. In pepper, *CaBBX4*, *CaBBX5*, and *CaBBX6* may positively participate in drought responses [24]. In maize, *ZmDBB2* positively regulates shade avoidance responses and plant height [61]. In potato, *StBBX14* positively regulates cold tolerance [37]. In sweet potato, *IbBBX24* positively regulates JA-mediated fungal resistance [73]; in yam, *DoBBX8/DoCOL8* positively regulates tuber formation under short-day and dark conditions [56]. Additionally, *NtBBX11* exerts positive regulatory functions under cold conditions [39]. The C-terminal region is a key determinant of positive versus negative regulatory functions. Through domain-swapping experiments, the C-terminal regions of *BBX24* (a negative regulator) and BBX21 (a positive regulator) have been shown to specify their opposite functions, whereas their B-box domains are interchangeable [8]. The B-box domains mediate the interaction with the bZIP domain of HY5, while the structurally divergent C-terminal regions determine whether a BBX protein acts as a positive or a negative regulator [8]. In addition, a predicted transactivation domain is present in *BBX20* and *BBX21*, and a 33-amino-acid fragment of *BBX21* containing this domain was shown to strongly activate transcription in yeast [70].

Negative regulators identified in *Arabidopsis* include *AtBBX18*, which negatively regulates heat tolerance [74]; *AtBBX24*, which antagonizes HY5 function through interaction, negatively regulating photomorphogenesis and UV-B responses [62]; *AtBBX25*, which similarly antagonizes HY5 function through interaction but negatively regulates photomorphogenesis and hypocotyl shade avoidance response [46]; and *AtBBX21*, which negatively regulates ABI5 expression and is required for normal seed germination under ABA [17], and which negatively regulates shade avoidance under long-term shading [57]. In tomato, *SlBBX18* functions as a negative regulator of drought tolerance by repressing APX1. The full-length *SpBBX18^CC^* protein inhibits APX1 transcription and enzymatic activity, whereas the protein encoded by the truncated *SlBBX18^TT^* allele loses the C-terminal transcriptional repression of APX1 but still reduces its enzymatic activity, leading to drought sensitivity [18]. In chrysanthemum, *CmBBX19* negatively regulates drought tolerance [33]; in rice, *OsBBX6* negatively responds to drought and salt stress [29], and *Ghd2* negatively regulates drought tolerance and accelerates drought-induced leaf senescence [31]. In sweet potato, *IbBBX28* negatively regulates drought tolerance [21]; in yam, *DoBBX2/DoCOL5* exhibits complex regulatory patterns during tuber formation: overexpression accelerates tuber formation under short-day conditions but shows the opposite effects under dark conditions [56]. Furthermore, *NtBBX9* and *NtBBX46* exhibit negative regulatory functions under cold stress [39,40].

Notably, some BBX proteins act bidirectionally: AtBBX28 and AtBBX29 function as negative regulators of photomorphogenesis [59], yet AtBBX28 positively regulates shade avoidance responses by promoting hypocotyl elongation [60]. Similarly, AtBBX21 positively regulates flowering and early shade-avoidance gene expression but negatively regulates ABA-responsive ABI5 expression and long-term shade avoidance [17,57].

The positive and negative regulatory functions of BBX proteins in diverse plant species are listed in Table 1.

## 4. Perspectives

Current research predominantly focuses on single stresses, but in natural environments, drought, cold, high light, and other stresses often occur simultaneously or sequentially [76]. Studies have shown that when six abiotic stresses are simultaneously applied to *Arabidopsis* seedlings, plant growth and survival rates decline sharply with increasing numbers of stress factors [76]. As key hubs connecting light signals to multiple stress responses, the integrated regulatory mechanisms of BBX proteins under multi-stress co-occurrence warrant in-depth investigation, including deciphering the interaction networks and functional redundancy among BBX members and their integrated responses under complex field conditions [77,78].

Structure–function relationships of BBX proteins. Although the C-terminus and VP motif have been identified as critical for functional differentiation, the complete map of key amino acids determining positive versus negative regulatory activity remains incomplete [77]. Phylogenetic analysis of BBX proteins across the green plant lineage revealed that B-box domains have undergone dynamic gains and losses, while the CCT domain is generally highly conserved across plant taxa, although secondary loss of the CCT domain has also been observed in certain lineages [79]. Combining structural biology with site-directed mutagenesis to dissect the structure–activity relationships of B-box, CCT, and C-terminal domains would provide a molecular basis for functional engineering [77,78].

Non-coding RNA regulatory networks targeting BBX. Although miRNAs have been predicted to target BBX genes, their regulatory roles under stress conditions remain largely unexplored. Whether long non-coding RNAs, circular RNAs, and other non-coding RNAs participate in stress responses through regulation of BBX expression remains largely unknown. Further experimental validation is needed to determine whether miRNAs regulate BBX expression under stress conditions, along with their specific underlying mechanisms.

Post-translational modifications of BBX proteins. Beyond the classical COP1-mediated ubiquitination pathway, BBX proteins in multiple plant species are subject to ubiquitination modifications by other E3 ligases: MdMIEL1 mediates ubiquitination and degradation of apple MdBBX37, thereby attenuating the cold resistance enhanced by MdBBX37 [42], and MdMIEL1 also targets *MdBBX7* [27]. MdBT2 mediates the ubiquitination and degradation of MdBBX22 [68]; additionally, the E3 ubiquitin ligase MdSINA3 targets MdBBX37 for degradation, negatively regulating JA-triggered leaf senescence [79]. SlMPK1/2-mediated phosphorylation of *SlBBX17* positively regulates CBF-dependent cold tolerance [34]. Post-translational modifications also involve other types such as phosphorylation and SUMOylation [80]. Their post-translational regulation remains poorly understood, and how it affects BBX stability, nuclear localization, or transcriptional activity represents an important frontier [77].

The C-terminal sequence is a key region determining positive versus negative regulatory functions. Through domain-swapping experiments, it has been demonstrated that swapping the C-terminus of AtBBX24 (negative regulator) with that of AtBBX21 (positive regulator) reversed their functions [8]. The C-terminus of *SpBBX18^CC^* (the wild-species allele, encoding SpBBX18) possesses transcriptional repression activity, whereas *SlBBX18^TT^* (the cultivated allele, encoding the truncated SlBBX18) lacks the C-terminus due to premature termination, losing the ability to repress APX1 transcription, but its N-terminus still reduces APX1 enzymatic activity, leading to drought sensitivity [18]. This finding provides strong evidence for the important role of the C-terminus in functional differentiation. In tobacco, sequence logo analysis revealed that the B-box1 domain is more conserved than B-box2 at certain positions (such as alanine at positions 9, 15, 18, and 35), while arginine (R), lysine (K), and tyrosine (Y) residues in the CCT domain are highly conserved [25], which may help maintain BBX protein structure and function.

Functional characterization of BBX genes in crops and molecular design breeding. Through CRISPR/Cas9 gene editing and haplotype analysis, the functions of BBX genes have been successfully validated in crops. For instance, CRISPR-Cas9-mediated knockout of *SlBBX18* in tomato revealed its negative regulatory role under drought stress [18]. Directed modification or stacking of identified positive regulators and negative regulatory domains is expected to enhance broad-spectrum resistance of crops to multiple stresses [77,78]. Investigating *AtBBX8*, which positively regulates freezing tolerance in a mainly CBF-independent manner [9], holds potential value for crop stress improvement, including provision of targets for multi-gene pyramiding breeding and utilization of light-responsive elements in its promoter for designing stress-inducible expression vectors. In summary, deciphering the regulatory networks and functional redundancy of BBX proteins will consolidate their role as central hubs of stress signaling and provide new targets for stress-resistant breeding [78].

## 5. Conclusions

In conclusion, the BBX family represents a central regulatory node that integrates light signals with abiotic stress responses in plants. Evidence from *Arabidopsis* and multiple crop species demonstrates that BBX proteins function as both positive and negative regulators in drought, cold, and UV-B responses, as well as in photomorphogenesis and shade avoidance. Emerging evidence also points to their involvement in heat and salt stress responses. This bidirectional regulation is structurally encoded by the number and type of B-box domains, by the presence of CCT and VP motifs, and especially by C-terminal sequence divergence. BBX proteins act through conserved modules involving HY5, COP1, PIFs, DELLA, ABF3, ICE1/CBF, and other partners, and their activities are further modulated by ubiquitination, phosphorylation, and natural allelic variation. In crops, *BBX* genes regulate stress tolerance, flowering time, plant height, tuber formation, anthocyanin biosynthesis, and disease resistance, demonstrating their potential for molecular design breeding. Future research should focus on multi-stress combinations, post-translational modifications, non-coding RNA regulation, and structure-guided engineering of BBX proteins. Such efforts will provide new targets for developing climate-resilient, high-yield crops.

## Figures and Tables

**Table 1:** Positive and negative regulatory functions of BBX proteins in diverse plant species.

Species	Gene/Protein	Type of Evidence	Positive Regulatory Function	Negative Regulatory Function	References
*Arabidopsis*	*AtBBX1* (CO)	Functional validation	Flowering		[15]
	*AtBBX5*	Functional validation	Salt/osmotic stress tolerance; UV-B resistance via anthocyanin biosynthesis		[16,64]
	*AtBBX7, AtBBX8*	Functional validation	Cold tolerance (mainly CBF-independent)		[9]
	*AtBBX9*	Functional validation	Photomorphogenesis		[43]
	*AtBBX20*	Functional validation	Photomorphogenesis, anthocyanin synthesis		[70]
	*AtBBX21*	Functional validation	Photomorphogenesis, anthocyanin synthesis [70]	① Negative regulation of ABI5 (ABA-hypersensitive germination); [17] ② Negative regulation of shade avoidance under long-term shading [57]	[17,57,70]
	*AtBBX22*	Functional validation	Photomorphogenesis, anthocyanin synthesis, photoprotection, and DNA repair under UV-B		[65,70]
	*AtBBX23*	Functional validation	Photomorphogenesis		[47]
	*AtBBX24*	Functional validation		Photomorphogenesis and UV-B responses	[62]
	*AtBBX25*	Functional validation		Photomorphogenesis; hypocotyl shade avoidance response	[46]
	*AtBBX18*	Functional validation		Thermotolerance	[74]
	*AtBBX28*	Functional validation	Shade avoidance	Photomorphogenesis	[59,60]
	*AtBBX29*	Functional validation		Photomorphogenesis	[59]
	*AtBBX31*	Functional validation	Positive regulation of UV-B signaling (under UV-B)	Photomorphogenesis (under white light)	[71]
*Tomato*	*SlBBX4*	Functional validation	Photomorphogenesis		[49]
	*SlBBX7*	Functional validation	Cold tolerance		[36]
	*SlBBX9*	Functional validation	Cold tolerance		[36]
	*SlBBX17*	Functional validation	Cold/heat stress		[34,72]
	*SpBBX18^CC^* (full-length)	Functional validation		Negative regulation of drought tolerance (maintains ROS homeostasis)	[18]
	*SlBBX18^TT^* (truncated)	Functional validation		Reduced drought tolerance (ROS accumulation)	[18]
	*SlBBX20 (Solyc12g089240)*	Functional validation	Photomorphogenesis/light signaling; flavonoid biosynthesis with SlHY5		[50]
	*SlBBX20,21*	Functional validation	Positive regulation of UV-B signaling		[67]
	*SlBBX26*	Functional validation	Vegetative growth, flowering and fruit development via SlPIF4 modulation		[51]
	*SlBBX31*	Functional validation (natural allelic variation)	Cold tolerance (natural allelic variation)		[35]
*Apple*	*MdBBX1*	Heterologous overexpression (in *Arabidopsis*)	Drought tolerance		[26]
	*MdBBX7*	Functional validation	Drought tolerance		[27]
	*MdBBX20*	Functional validation	Low-temperature-induced anthocyanin accumulation		[41]
	*MdBBX21*	Heterologous overexpression (in *Arabidopsis*) + apple calli	Light-induced anthocyanin accumulation		[54]
	*MdBBX22*	Functional validation	UV-B-induced anthocyanin biosynthesis	Negative regulation of ABA-induced leaf senescence	[28,68]
	*MdBBX37*	Functional validation	Cold tolerance	degraded by MdSINA3, attenuating JA-triggered leaf senescence	[42,75]
*Rice*	*OsBBX6*	Expression data only (candidate)		Negative response to drought and salt stress	[29]
	*OsBBX11*	Functional validation	Na^+^/K^+^ transport; salt tolerance		[32]
	*OsBBX14*	Functional validation	Photomorphogenesis under blue light		[52]
	*Ghd2 (OsBBX8)*	Functional validation		Drought sensitivity, accelerated leaf senescence	[31]
*Pepper*	*CaBBX4,5,6*	Expression data only (candidate)	Drought response (putative)		[24]
*Maize*	*ZmDBB2*	Functional validation	Shade avoidance, plant height		[61]
*Potato*	*StBBX14*	Functional validation	Cold tolerance		[37]
*Sweet potato*	*IbBBX24*	Functional validation	JA-mediated fungal resistance		[73]
	*IbBBX28*	Heterologous overexpression (in *Arabidopsis*)		Negative regulation of drought tolerance	[21]
*Yam*	*DoBBX8/DoCOL8*	Heterologous overexpression (in potato)	Tuber formation under short-day and dark conditions		[56]
	*DoBBX2/DoCOL5*	Heterologous overexpression (in potato)		Reduced tuber formation relative to DoBBX8 under dark	[56]
*Chrysanthemum*	*CmBBX19*	Functional validation		Negative regulation of drought tolerance	[33]
*Tobacco*	*NtBBX11*	Functional validation	Cold stress		[39]
	*NtBBX9,46*	Functional validation		Negative regulation of cold stress	[39,40]

## Data Availability

No new data were created or analyzed in this study. Data sharing is not applicable to this article.
